# Statistics of Medical Colleges

**Published:** 1859-04

**Authors:** 


### STATISTICS OF MEDICAL COLLEGES FOR THE SESSION 1858-9.

The following statistical table includes all the schools we have heard from.

It is necessarily incomplete; but we shall keep the table standing, and endeavor to complete it in the May number.

If errors occur, we shall be glad to be corrected from any quarter.

| | Students. | Graduates. |
|---|---|---|
| College of Physicians and Surgeons, (N. Y.) | 180 | 39 |
| University of New York, . . . . . | 350 | 128 |
| New York Medical College, . . . . | 107 | 25 |
| University, Buffalo, . . . . . . | 67 | 13 |
| Albany Medical College, . . . . . | — | 48 |
| Geneva Medical College, . . . . . | — | — |

| | Students. | Graduates. |
|---|---|---|
| Jefferson Medical College, . . . . | 570 | 256 |
| University of Pennsylvania, . . . . | 410 | 140 |
| Pennsylvania Medical School, . . . | 150 | 33 |
| Philadelphia School of Medicine, . . | 130 | 17 |
| Pennsylvania University, . . . . | — | — |
| Rush Medical College, . . . . . | 127 | 31 |
| University, Louisiana, . . . . . | 306 | — |
| New Orleans School of Medicine, . . | 140 | 36 |
| Missouri Medical College, . . . . | — | 23 |
| St. Louis Medical College, . . . . | 135 | 40 |
| Massachusetts Medical College, . . . | 139 | 30 |
| University of Louisville, . . . . . | — | 35 |
| Kentucky School of Medicine, . . . | 103 | 28 |
| Cleveland Medical College, . . . . | — | — |
| Ohio Medical College, . . . . . | 140 | 32 |
| Oglethorpe Medical College, . . . . | 50 | 13 |
| Savannah Medical College, . . . . | 34 | 8 |
| University of South Carolina, . . . | — | — |
| Memphis Medical College, . . . . | — | — |
| Shelby Medical College, . . . . . | 53 | 11 |
| Medical College of Virginia, . . . . | — | 20 |
| University of Virginia, . . . . . | — | — |
| University of Maryland, . . . . . | — | — |
| Yale Medical College, . . . . . | — | — |
| Castleton Medical College, . . . . | — | — |
| Berkshire Medical College, . . . . | — | — |
| University of Iowa, . . . . . . | — | — |
| Woodstock Medical College, . . . . | — | — |
| Medical College of Georgia (Augusta) . | 165 | 58 |
| Dartmouth Medical College, . . . . | — | 9 |
| Atlanta Medical College, . . . . . | — | — |
| University of Michigan, . . . . . | 143 | — |
| Starling Medical College, . . . . . | — | — |
| University, Vermont, . . . . . . | — | — |
| University, Nashville, . . . . . | 436 | 103 |
| Medical College of Maine, . . . . | 50 | — |

---

UNIVERSITY OF THE PACIFIC.

A new medical school opens in May next, at San Francisco, California. Its present faculty consists of Drs. Morrison, Rowell, Cole, and Cooper. Dr. Cooper formerly resided at Peoria, Ill.

